# Assessment of Natural Occurrence and Risk of the Emerging Mycotoxin Moniliformin in South Korea

**DOI:** 10.3390/toxins17020050

**Published:** 2025-01-23

**Authors:** So Young Woo, Sang Yoo Lee, Su Been Park, Si Eun Kim, Young Woon Kang, Hyang Sook Chun

**Affiliations:** 1 GreenTech-Based Food Safety Research Group, BK21 Four, School of Food Science and Technology, Chung-Ang University, Anseong-si 17546, Republic of Korea; mochalatte9@naver.com (S.Y.W.); dm3822@naver.com (S.Y.L.); sirius6100@naver.com (S.B.P.); 2 Department of Food Safety and Regulatory Science, Chung-Ang University, Anseong-si 17546, Republic of Korea; 3 Food Contaminants Division, National Institute of Food and Drug Safety Evaluation, Ministry of Food and Drug Safety, Cheongju-si 28159, Republic of Korea; youngcloud@korea.kr

**Keywords:** moniliformin, emerging mycotoxin, risk assessment

## Abstract

Moniliformin (MON) is a highly polar, emerging *Fusarium* mycotoxin with a low molecular weight. It is known to exhibit potentially harmful effects on public and animal health. This study aimed to comprehensively assess the natural occurrence of MON in various foods marketed in South Korea and to perform a risk assessment. An analytical method for MON quantification using strong anion exchange clean-up combined with ultra-high-performance liquid chromatography–tandem mass spectrometry was validated across four different food matrices (white rice, sorghum, corn oil, and baby food), exhibiting excellent accuracy, precision, and sensitivity. A total of six food categories, 33 food commodities, and 253 food samples were included in this study. Maize, sorghum, Job’s tears, and perilla seeds were identified as the major contributors to MON contamination. Estimated daily intake (EDI) was calculated for both mean and 95th percentile extreme dietary scenarios using upper and lower bound approaches. The highest EDI was observed in the 0–2-year and 3–6-year age groups, primarily for cereal grains. The margin of exposure (MOE) values for maize consumption ranged from 2544 to 7482. These results highlight the potential health concerns associated with MON, necessitating targeted risk management strategies.

## 1. Introduction

*Fusarium* mycotoxins are among the most diverse and common mycotoxins, posing significant challenges to food safety and public health [1]. Recent attention has focused on emerging mycotoxins, which are not yet regulated or routinely monitored but may have considerable health implications. Moniliformin (MON) is a widespread emerging mycotoxin produced by various *Fusarium* species, including *F. avenaceum*, *F. subglutinans*, *F. proliferatum*, and *F. fujikuroi* [2,3].

MON mainly exists in nature as the sodium or potassium salt of 3-hydroxy-3-cyclobutene-1,2-dione, also known as semisquaric acid [4]. Its small size, high polarity, and strong acidity (pKa 0.05–1.7) pose analytical challenges. As MON is weakly retained in reversed-phase chromatography, its detection and quantification are complicated [5,6,7]. Traditional methods, such as high-performance liquid chromatography (HPLC) with diode array UV detection, have been used for quantification; however, their high detection limits (41–80 μg/kg) hinder accurate quantification of the natural occurrence of MONs and their associated risks [4,5,8]. Although liquid chromatography–tandem mass spectrometry (LC–MS/MS) exhibits improved sensitivity and specificity, research on its efficiency in diverse food matrices remains limited.

MON is extremely toxic to various animals, including chickens, rats, swine, and ducks [9,10]. MON exposure has been associated with cardiotoxic effects, such as myocardial degeneration and ventricular hypertrophy, as well as hematotoxicity [11,12]. It inhibits enzyme systems, thereby blocking the entry of pyruvate into the Krebs cycle, reducing mitochondrial respiration, and ultimately disrupting cellular energy production [3,13]. Additionally, MON exposure resulting from high corn consumption has been suspected to cause a heart condition known as Keshan disease in humans in China and South Africa [14,15].

The acute reference dose (ARfD) and tolerable daily intake (TDI) of MON could not be established because of limitations in available toxicity data. Hence, the EFSA (European Food Safety Authority) Panel on Contaminants in the Food Chain (CONTAM) assessed risks using the margin of exposure (MOE) approach [12]. For acute risk, the MOE was calculated by comparing the no-observed-adverse-effect level (NOAEL) of 6.0 mg/kg b.w., derived from a subacute study on rats, with the acute upper bound (UB) dietary exposure estimates [12]. For chronic risk, the MOE was calculated by comparing the lowest benchmark dose lower confidence limit for a 5% response (BMDL_05_) of 0.20 mg MON/kg b.w./day, calculated for hematological hazards in a 28-day study on pigs, with the chronic dietary human exposure estimates [12].

Despite the global concern surrounding emerging mycotoxins, there have been no comprehensive studies reporting the occurrence and distribution of MON in foods marketed in South Korea. Therefore, in this study, we developed an analytical method for the precise determination of MON in food products using strong anion exchange (SAX) solid-phase extraction (SPE) purification with ultra-high-performance liquid chromatography (UPLC)–electrospray ionization (ESI)–triple quadrupole (QqQ) MS. We used this validated method to comprehensively assess the contamination levels of MON in foods marketed in South Korea. Moreover, we assessed potential dietary exposure to determine associated risks.

## 2. Results and Discussion

### 2.1. Performance of the Analytical Method

The sample preparation method was optimized in the white rice matrix by considering the extraction solvent and the clean-up process, including SPE cartridges, as well as the loading and washing solvents for the cartridges (Figure S1). Methanol (MeOH)-based extraction solvents (75–93%) showed higher recovery than acetonitrile (ACN)-based solvents (27–91%). Finally, 85% MeOH (*v*/*v*), which exhibited the highest recovery rate, was selected as the extraction solvent. The purification performance of four different SPE cartridges was evaluated. The Bond Elut SAX column, which showed the highest recovery rate (93–121%), was selected for further optimization of the loading solvent and the washing method. In most previous studies using SAX SPE, the entire supernatant of the extract was dried, followed by reconstitution in 100% MeOH before loading onto the cartridge [6,16,17]. This process increases the time required for sample preparation and leads to a loss of analyte during drying [18,19]. The extraction solvent (85% MeOH, v/v), which showed the highest recovery, exhibited excellent performance even when applied directly to the cartridge without a drying step. Therefore, the extracted supernatant was directly loaded onto the SAX column without additional drying. The cartridge washing method involved the use of 50% MeOH (*v*/*v*), followed by water. The aim was to effectively remove the food matrix while minimizing the loss of MON bound to the column. The final optimized analytical method is described in detail in Section 4.4.

**Figure 1:**
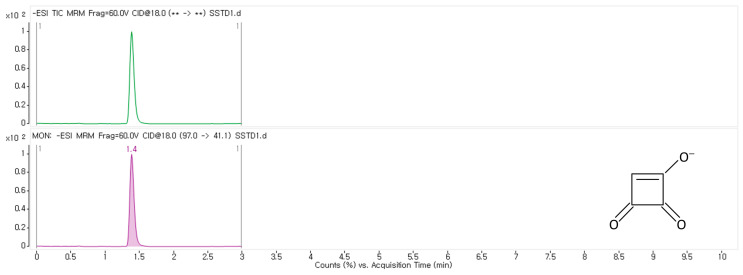
Chromatograms of MON at 1 μg/kg in sorghum matrix (top: TIC, bottom: MRM).

Method validation was performed using four different food matrices—white rice, sorghum, corn oil, and baby food—selected based on EC No. 519/2014 [20]. Grain products are the most frequently reported food group contaminated with MON [2,6,19]. Among these, white rice is the most consumed grain in South Korea. It serves as a representative matrix for high-starch, low-water, and low-fat foods. Sorghum, known for its high protein content and strong pigmentation, is a grain frequently associated with various *Fusarium* mycotoxins [21,22]. The corn oil matrix was chosen as a representative for food commodities characterized by high oil and very low water contents. Additionally, as infants and young children are particularly vulnerable to mycotoxin exposure [23], cereal-based baby foods were chosen as a validation matrix to ensure accurate analysis and enable precise assessment of natural occurrence and risk. The representative chromatograms of MON (1 μg/kg) in the sorghum matrix are shown in Figure 1, and the validation results in four food matrices are presented in Table 1. Linearity in all matrices was excellent, with coefficient of determination (R^2^) values exceeding 0.99. The limit of detection (LOD) ranged from 0.04 to 0.07 µg/kg, and the (limit of quantification) LOQ ranged from 0.11 to 0.22 µg/kg, confirming the applicability of the method to perform quantification at levels lower than those in previous studies [4,6,8,24]. For all matrices, the accuracy was between 90.2% and 109.6%, all obtained results satisfying the Association of Official Analytical Chemists (AOAC) Guidelines for Standard Method Performance Requirements (SMPRs) [25]. The within-laboratory repeatability (RSD_r_) and inter-laboratory reproducibility (RSD_R_) were 1.8–14.2% and 4.6–12.7%, respectively. The measurement uncertainty was calculated to be less than 8.7%, which is acceptable according to the 2004 EC report, stating that the expanded uncertainty should be less than 44% for analyte concentrations below 100 μg/kg [26]. The validated method was used to investigate the natural occurrence of MON in commercially available foods in South Korea.

### 2.2. Natural Occurrence of MON

The natural occurrence of MON was assessed in 253 commercial foods in South Korea (Table 2). Among cereal grains, 64% (68/107) of the samples were contaminated; the mean contamination level was 49.19 μg/kg (range: 0.94–374.10 μg/kg). The incidence of positive samples was highest in Job’s tears (100% of the tested samples), followed by sorghum (93%), brown rice (87%), maize (80%), white rice (67%), foxtail millet (60%), and black rice (33%). The mean contamination level was highest in sorghum (153.31 μg/kg), followed by maize (100.80 μg/kg), Job’s tears (97.19 μg/kg), foxtail millet (31.34 μg/kg), brown rice (4.14 μg/kg), black rice (2.67 μg/kg), and white rice (1.16 μg/kg). The maximum contamination level was detected in sorghum (374.10 μg/kg), while no contamination was detected in barley or oats.

Among processed grain products, MON was detected in 56% (23/41) of the samples; the mean contamination level was 5.05 μg/kg (range: 0.94–38.72 μg/kg). MON was most prevalently detected in wheat flour (100%), followed by Sunsik (a cereal-based ready-to-drink powder) (82%) and popcorn maize (80%). The mean contamination level was highest in popcorn maize (12.57 μg/kg), followed by Sunsik (11.63 μg/kg) and wheat flour (1.62 μg/kg). The maximum contamination level was detected in Sunsik (38.72 μg/kg), while no contamination was detected in canned maize or cereal-based baby foods.

In pulses, the incidence of MON was low at 18%, with contamination levels ranging from 1.45 to 7.63 μg/kg. MON was detected at 1.45 μg/kg in one mung bean sample and at 7.63 μg/kg in one black bean sample. Among seasoning foods, 30% (6/20) of the samples were contaminated with MON; the mean contamination level was 0.38 μg/kg (range: 0.06–4.18 μg/kg). Nutmeg had the highest contamination at 60%, followed by turmeric (40%) and curry powder (20%). However, the contamination levels in positive samples were low (below 4.18 μg/kg for all). Among nuts and seeds, MON was detected only in peanut and perilla seed samples. A low level of contamination (9.93 μg/kg) was detected in a single peanut sample. However, MON was detected in 60% of perilla seed samples, with the contamination levels reaching up to 285.15 μg/kg. MON was not detected in any of the 46 edible oil samples.

Our investigation of the natural occurrence of MON revealed that maize, sorghum, Job’s tears, and perilla seeds are the primary sources of MON contamination in foods marketed in South Korea. Maize is one of the most well-known sources of MON contamination. The maximum contamination level in maize samples marketed in South Korea was 256.5 μg/kg, which was lower than the levels reported previously worldwide (average contamination levels ranging from 34.4 to 2255 μg/kg and maximum levels ranging from 1742 to 4800 μg/kg) [27,28,29]. However, the positive detection rate of 80% observed in this study was comparable to or slightly higher than the rates reported globally (ranging from 47% to 100%) [5,27,29]. Only a few studies have reported MON contamination in sorghum, with the mean contamination levels ranging from 58.0 to 605.1 μg/kg, maximum levels ranging from 437.0 to 914.2 μg/kg, and positive incidence rates ranging from 41% to 93% [30,31]. Notably, to the best of our knowledge, this is the first study to report MON contamination in Job’s tears and perilla seeds. Their contamination levels and detection rates were comparable to those in maize, highlighting the need for further research and focus on these food groups. In contrast, MON was not detected in barley and oats in this study, despite previous reports stating that these grains are frequently associated with the occurrence of MON [7,12].

### 2.3. Risk Assessment of MON 

The calculated estimated daily intake (EDI) and margin of exposure (MOE) values are presented in Table 3. The EDI for the total population from mean dietary intake was highest for cereal grains, ranging from 1.2902 (lower bound, LB) to 1.2986 ng/kg b.w./day (upper bound, UB), followed by processed grain products, pulses, seasoning foods, and edible oils. The lowest EDI was observed for seasoning foods, ranging from 1.411 × 10^−4^ (LB) to 6.600 × 10^−4^ ng/kg b.w./day (UB).

**Figure 2:**
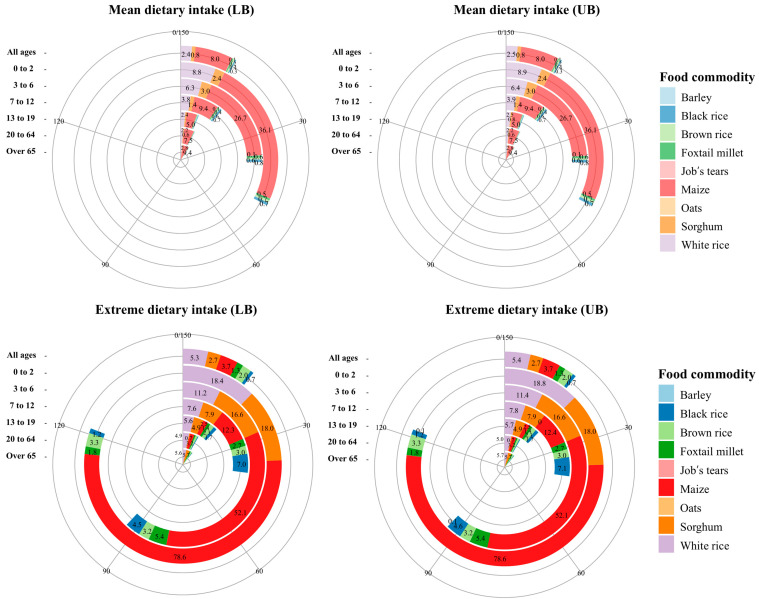
Contribution of each cereal grain to mean and extreme (95th percentile) dietary exposures to MON. Each segment represents an age group, denoting groups from all ages (outermost) to over 65 years (innermost). The length of each colored bar indicates the EDI (ng/kg b.w./day) contributed by the consumption of each cereal grain.

The highest dietary exposure from cereal grains was noted in the 0–2-year age group, followed by the 3–6-year age group. The contribution of individual food commodities to the EDI is illustrated in Figure 2, focusing on cereal grains that accounted for the majority of the exposure. Assessment of the contribution to the EDI (LB) for the mean dietary scenario in the total population revealed that maize was the highest contributor at 61.8% (UB, 61.4%), followed by white rice at 18.6% (UB, 18.9%) and sorghum at 6.0% (UB, 5.9%). Although sorghum exhibited the highest contamination levels, the higher consumption of white rice and maize resulted in their greater EDI values. For the extreme dietary scenario, white rice, which had the highest consumption rate, contributed the most to the EDI at 29.1% (UB, 29.3%), followed by maize at 20.3% (UB, 20.1%) and sorghum at 14.9% (UB, 14.8%). In this scenario, brown rice, Job’s tears, perilla seeds, and wheat flour were also identified as significant sources, with each accounting for more than 5% of the total EDI. Additionally, our results revealed that the differences between the LB and UB scenarios are minimal, primarily due to the low LOD values (0.04–0.07 μg/kg) and the high proportion of occurrence data above the LOD.

According to the guidance of the EFSA Scientific Committee, an MOE value exceeding 10,000 is considered to indicate low safety concerns [32]. In our study, the MOE calculated as per food category was above 10,000 in all age groups for both the lower bound (LB) and upper bound (UB) scenarios. However, when examined as per individual food commodities, certain MOE values fell below 10,000. In all scenarios, the MOE values for maize consumption in the 0–2-year and 3–6-year age groups ranged from 2544 to 7482, highlighting a significant public health concern. The finding that the MOE values for the 0–2-year and 3–6-year age groups were below 10,000 is particularly significant because infants and young children are the most vulnerable populations to mycotoxins worldwide [33]. The diets of infants and young children are predominantly composed of cereal-based complementary foods [34]. This dietary characteristic, combined with their lower body weight, contributes to higher MON exposure. To date, limited research has been conducted on the risk of MON through food consumption. The EFSA CONTAM Panel reported that the mean chronic exposure to MON ranged from 0.04 to 51 ng/kg b.w./day (LB) and 51–226 ng/kg b.w./day (UB), while the MOE values for the mean dietary scenario ranged from 3900 to 5,000,000 (LB) and 880–25,000 (UB) [12]. Dietary exposure to MON from foods marketed in South Korea and MOE values were confirmed to be relatively low, with the maximum dietary exposure being 36.14 ng/kg b.w./day and minimum MOE being 2544 for the extreme dietary scenario based on UB approaches.

## 3. Conclusions

In this study, an analytical method for MON quantification was developed and validated, focusing on improving recovery and precision across diverse food matrices. Additionally, a comprehensive investigation of MON occurrence in foods marketed in South Korea was performed, calculating dietary exposure and MOE values across age groups. MON contamination was predominantly observed in cereal grains, with maize, sorghum, and Job’s tears identified as the major contributors to dietary exposure. The incidence of positive samples and contamination levels in perilla seeds and Job’s tears were notable, marking this as the first study to report MON contamination in these commodities. In contrast, barley and oats, which have frequently been associated with MON contamination in previous studies, were not found to be contaminated with MON in this study.

Risk assessments revealed that the mean EDI was highest for cereal grains in all age groups, especially in infants and young children (0–2 and 3–6 years, respectively). The MOE values for maize consumption in these age groups ranged from 2544 to 7482, falling below the critical threshold of 10,000. This indicates a potential public health concern, especially given the heightened vulnerability of children to mycotoxins because of their lower body weight and cereal-based diets.

This study is the first to report the natural occurrence of MON in various foods marketed in South Korea. Our findings emphasize the need for continued monitoring of MON in high-risk food commodities and susceptible populations.

## 4. Materials and Methods

### 4.1. Sample Collection

A total of 253 commercial food products distributed in South Korea were purchased from both online retailers and local markets between 2022 and 2023. Of these, 191 samples were obtained from online retailers to ensure access to a diverse range of brands and processed products, reflecting recent consumer purchasing trends, while 62 samples were collected from local markets to provide a comprehensive representation of products available nationwide. The collected samples were classified into the following food categories: cereal grains (white rice, brown rice, black rice, barley, oats, Job’s tears, sorghum, maize, and foxtail millet), processed agricultural products (wheat flour, baby food, Sunsik, canned maize, and popcorn maize), pulses (red bean, mung bean, and black bean), seasoning foods (red pepper powder, curry powder, turmeric, and nutmeg), nuts and seeds (peanut, walnut, almond, sesame seed, and perilla seed), and edible oils (corn oil, sesame oil, perilla oil, red pepper seed oil, grapeseed oil, rapeseed oil, olive oil, and soybean oil). Each sample was obtained in a quantity of at least 1 kg, homogenized, and stored at −20 °C. Before analysis, the frozen samples were equilibrated to room temperature.

### 4.2. Chemicals and Reagents

A certified MON standard was obtained from Romer Labs (Tulln, Austria), with a certified mass concentration of 100.2 µg/mL and purity of 97.0% ± 1.0%. A working standard was prepared in ACN at a concentration of 1 µg/mL. HPLC-grade water, ACN, and MeOH were obtained from Honeywell Burdick & Jackson (Muskegon, MI, USA), and LC–MS-grade formic acid was purchased from Thermo Fisher Scientific (Bremen, Germany). The ion-pairing reagent for sample preparation was prepared using tetrabutylammonium hydroxide (TBAH) solution (40 wt.% in H_2_O, suitable for ion chromatography) obtained from Supelco (Bellefonte, PA, USA) and 1.0 M potassium phosphate monobasic solution obtained from Sigma-Aldrich (St. Louis, MO, USA).

### 4.3. UPLC–ESI–QqQ MS Equipment Conditions

UPLC–MS/MS analysis was performed using an Agilent 1290 Infinity II system coupled with an Agilent 6470 Triple Quadrupole mass spectrometer (Santa Clara, CA, USA). Chromatographic separation was achieved on a Supelco Ascentis Express C18 column (2.1 × 100 mm, 2.7 μm) with an Ascentis guard column (2.1 × 5 mm, 2.7 μm). The mobile phase consisted of solvent A (water with 0.1% formic acid, *v*/*v*) and solvent B (MeOH with 0.1% formic acid, *v*/*v*). The gradient program began with the holding of 20% B for 3 min, followed by a linear increase to 90% B at 5.5 min, which was sustained until 7 min. At 7.1 min, the percentage of B was decreased to 20% and maintained until 10 min. The flow rate was 0.3 mL/min, and the injection volume was 2 μL. The column temperature was set to 40 °C. The mass transition for MON was monitored at 97.0 *m*/*z* → 41.1 *m*/*z*, which corresponds to a single product ion. The fragmentor voltage was set to 60 V, with a collision energy of 18.0 V. The retention time of the analyte was 1.4 min. MS detection was performed in negative ESI mode. The gas flow rate was 9 L/min, and the nebulizer pressure was 30 psi. The sheath gas temperature and flow rate were set to 350 °C and 12 L/min, respectively, while the drying gas temperature was 250 °C. The nozzle voltage was 0 V, and the capillary voltage was −2000 V. Ultrapure nitrogen (>99.99%) was used as the collision gas.

### 4.4. Sample Preparation and Method Validation

A homogenized sample (5 g) was extracted with 25 mL of extraction solvent (85% MeOH, *v*/*v*) by shaking at 270 rpm for 30 min, followed by centrifugation at room temperature at 3100× *g* for 5 min. Clean-up was performed using SAX SPE (Bond Elut SAX, 500 mg, 6 cc; Agilent Technologies, Santa Clara, CA, USA). The SAX cartridge was sequentially preconditioned with 2 mL of MeOH, 2 mL of water, and 2 mL of 0.1 M HCl. After preconditioning, 5 mL of the supernatant was loaded onto the cartridge at a flow rate of 1 drop/s. The cartridge was washed with 2 mL of 50% MeOH (*v*/*v*), followed by 2 mL of water. Subsequently, a vacuum pump was used to dry the cartridge for 3 min. MON was eluted from the cartridge with 2 mL of an ion-pairing reagent, prepared by mixing 150 mL of HPLC-grade water with 50 mL of 20% TBAH and 100 mL of 1.0 M potassium phosphate monobasic solution. The eluate was collected under pressure to ensure complete passage through the cartridge, filtered using a 0.2 μm pore size PVDF syringe filter (13 mm), and subsequently injected into the UPLC–MS/MS system.

The performance of the method was assessed in accordance with the AOAC Guidelines for SMPRs [25]. Matrix-matched calibration curves ranging from 1 to 50 μg/kg were created by spiking MON into blank matrices. Linearity was determined based on R^2^ values obtained using six-point matrix-matched calibration curves, where the signal intensity was plotted against the MON concentration. LOD and LOQ were calculated based on the slope of the matrix-matched calibration curve (S) and the standard deviation (SD) of the peak area at the lowest calibration concentration point. The following formulas were used: LOD = 3.3 × SD/S and LOQ = 10 × SD/S. Accuracy was assessed through recovery experiments by using the following formula: measured concentration of spiked samples derived from matrix-matched standards/spiking concentration × 100 (%). Precision was evaluated as the RSD_r_ and RSD_R_ through inter-laboratory recovery experiments conducted in three different laboratories. Measurement uncertainty was calculated as per the EURACHEM/CITAC Guide CG 4—Quantifying Measurement Uncertainty in Analytical Measurement [35]. The sources of uncertainty included the reference material (purity of the standard and preparation of the working standard), sample preparation (sample weight and final volume), instrumental factors, the interpolation of the calibration curve, and matrix effects (recovery). The combined standard uncertainty was derived by taking the square root of the total variance from all identified sources of uncertainty. The expanded uncertainty was derived using a coverage factor of two, which gives a confidence level of approximately 95%.

### 4.5. Risk Assessment

Food consumption data and body weight by age group were obtained from the Korean National Health and Nutrition Examination Survey VIII (KNHANES, 2019–2021). The average weight for the 1–2 years group is 12.37 kg, followed by 19.37 kg for the 3–6 years, 38.78 kg for the 7–12 years, 61.56 kg for the 13–19 years, 66.85 kg for the 20–64 years, and 60.26 kg for the over 65 years. The overall average weight across all age groups is 61.32 kg. Food consumption data are presented in Table S1. An exposure assessment was performed using two distinct approaches, namely LB and UB approaches, to determine the mean EDI. In the LB approach, the EDI was calculated by considering a value of zero for all nondetected samples. In contrast, in the UB approach, the EDI was estimated by assigning LOD values, which ranged from 0.04 to 0.07 μg/kg depending on the matrix-matched calibration, to nondetected samples. For dietary consumption, two scenarios were considered: (1) mean dietary intake and (2) 95th percentile extreme dietary intake. Dietary exposure was determined based on the EDI (ng/kg b.w./day), which was calculated by multiplying the mean occurrence level of MON in food commodities (ng/kg) by the daily consumption of the relevant food commodity in each age group (kg/day) and dividing the result by the mean body weight of the population within the corresponding age group (kg) [Equation (i)]. The CONTAM Panel identified the lowest BMDL_05_ of 0.20 mg MON/kg b.w./day based on hematological adverse effects as an indicative reference point for assessing the risk of chronic dietary exposure to MON [12]. Using the BMDL_05_ value of 0.2 mg/kg b.w./day, the MOE was calculated by dividing the BMDL_05_ by the EDI (ng/kg b.w./day), as described in Equation (ii). Visualizations of dietary exposure data were performed using ggplot2 (version 3.5.1) in R (version 4.3.2).

$$(i)\;\mathrm{EDI}\;(\mathrm{ng}/\mathrm{kg}\;b.w./\mathrm{day})=\frac{{Occurrence\;level(ng/kg)}^{\;(1)}\times {Daily\;consumption\;(kg/day)}^{\;(2)}}{Mean\;body\;weight\;(kg)}\;(\mathrm{ii})\;\mathrm{MOE}=\frac{{BMDL}_{05}\;(200,000\;ng/kgb.w./day)}{EDI\;(ng/kgb.w./day)}$$

^(1)^ Occurrence level: LB (nondetected = 0) or UB (nondetected = LOD value) approach.

^(2)^ Daily consumption: Mean dietary intake or 95th percentile extreme intake by age group.

## Figures and Tables

**Table 1:** Performance of method used for quantifying MON in food matrices.

Matrix	Linearity (R^2^)	LOD ^(1)^ (μg/kg)	LOQ ^(2)^ (μg/kg)	Spiking Level (μg/kg)	Intra-Day (*n* = 9) (%)		Inter-Day (*n* = 9) (%)		RSD_R_^(4)^ (%)	Uncertainty/ Results (%)
					Recovery	RSD_r_	Recovery	RSD_r_^(3)^		
White rice	0.999	0.07	0.22	10	92.1	2.6	90.2	1.8	4.6	8.7
				20	91.7	6.1	93.9	4.4	5.1	5.3
				50	95.9	5.6	96.3	7.3	6.9	6.7
Sorghum	1.000	0.05	0.16	10	93.4	14.1	99.2	11.1	11.7	5.6
				20	101.7	8.6	102.6	6.2	10.1	6.2
				50	106.8	10.7	104.5	5.8	8.5	4.7
Baby food	0.999	0.04	0.11	10	104.7	2.2	98.3	9.4	7.2	6.9
				20	109.6	2.1	106.1	5.9	5.0	6.4
				50	96.0	3.3	93.0	5.0	5.9	5.7
Corn oil	0.997	0.04	0.11	10	92.5	9.7	103.4	14.2	12.7	4.8
				20	106.8	4.8	109.4	6.1	7.1	5.0
				50	104.1	8.9	104.2	9.7	8.5	4.3

^(1)^ Limit of detection, ^(2)^ limit of quantification, ^(3)^ within-laboratory repeatability, ^(4)^ inter-laboratory reproducibility.

**Table 2:** Occurrence of MON in food commodities (n = 253).

Food Category	Food Commodity	Incidence of Positive Samples ^(1)^	Occurrence (μg/kg)				
			Mean	Positive Mean	Minimum	Median	Maximum
Cereal grains	White rice	67%	1.16	1.74	1.02	1.67	3.52
	Brown rice	87%	4.14	4.78	0.94	1.77	34.78
	Black rice	33%	2.67	8.01	-	-	-
	Barley	0%	- ^(2)^	-	-	-	-
	Oats	0%	-	-	-	-	-
	Job’s tears	100%	97.19	97.19	18.40	85.74	248.47
	Sorghum	93%	153.31	164.26	0.99	131.83	374.10
	Maize	80%	100.80	126.00	12.74	123.30	256.50
	Foxtail millet	60%	31.34	52.24	1.08	22.50	192.45
Processed grain products	Wheat flour	100%	1.62	1.62	0.94	1.41	3.30
	Canned maize	0%	-	-	-	-	-
	Popcorn maize	80%	12.57	15.71	7.89	16.10	22.76
	Baby foods	0%	-	-	-	-	-
	Sunsik	82%	11.63	14.22	1.76	5.61	38.72
Pulses	Red bean	0%	-	-	-	-	-
	Mung bean	33%	0.48	1.45	1.45	-	-
	Black bean	20%	1.53	7.63	7.63	-	-
Seasoning foods	Red pepper powder	0%	-	-	-	-	-
	Curry powder	20%	0.07	0.33	0.33	0.00	0.33
	Turmeric	40%	0.17	0.42	0.41	0.42	0.44
	Nutmeg	60%	1.28	2.14	0.06	2.18	4.18
Nuts and seeds	Peanut	13%	1.24	9.93	9.93	-	-
	Walnut	0%	-	-	-	-	-
	Almond	0%	-	-	-	-	-
	Sesame seed	0%	-	-	-	-	-
	Perilla seed	60%	67.66	112.76	6.04	47.09	285.15
Edible oils	Corn oil	0%	-	-	-	-	-
	Sesame oil	0%	-	-	-	-	-
	Perilla oil	0%	-	-	-	-	-
	Red pepper seed oil	0%	-	-	-	-	-
	Grapeseed oil	0%	-	-	-	-	-
	Rapeseed oil	0%	-	-	-	-	-
	Olive oil	0%	-	-	-	-	-
	Soybean oil	0%	-	-	-	-	-
Total		41%	23.07	56.66	0.06	12.13	374.10

^(1)^ Number of positive samples/total number of samples × 100 (%), ^(2)^—means not detected (<LOD).

**Table 3:** Estimated dietary exposure to MON and MOE values for mean and extreme (95th percentile) dietary scenarios based on food category.

Age Group	Scenario		Cereal Grains		Edible Oils		Nut and Seeds		Processed Grain Products		Pulses		Seasoning Foods	
			EDI ^(1)^	MOE	EDI	MOE	EDI	MOE	EDI	MOE	EDI	MOE	EDI	MOE
All ages	Mean	LB	1.2902	1.6 × 10^5^	- ^(1)^	-	0.0608	3.3 × 10^6^	0.1015	2.0 × 10^6^	0.0682	2.9 × 10^6^	0.0001	1.4 × 10^9^
	Dietary	UB	1.2986	1.5 × 10^5^	0.0004	5.5 × 10^8^	0.0611	3.3 × 10^6^	0.1016	2.0 × 10^6^	0.0701	2.9 × 10^6^	0.0007	3.0 × 10^8^
	Extreme	LB	1.8869	1.1 × 10^5^	-	-	0.2388	8.4 × 10^5^	0.2909	6.9 × 10^5^	0.1655	1.2 × 10^6^	NA ^(2)^	NA ^(^^2)^
	Dietary	UB	1.9087	1.0 × 10^5^	0.0015	1.4 × 10^8^	0.2397	8.3 × 10^5^	0.2909	6.9 × 10^5^	0.1699	1.2 × 10^6^	0.0017	1.2 × 10^8^
0 to 2 years	Mean	LB	5.0925	3.9 × 10^4^	-	-	0.1188	1.7 × 10^6^	0.1255	1.6 × 10^6^	0.1111	1.8 × 10^6^	0.0004	4.7 × 10^8^
	Dietary	UB	5.1208	3.9 × 10^4^	0.0009	2.1 × 10^8^	0.1192	1.7 × 10^6^	0.1259	1.6 × 10^6^	0.1142	1.8 × 10^6^	0.0008	2.4 × 10^8^
	Extreme	LB	13.1299	1.5 × 10^4^	-	-	0.2346	8.5 × 10^5^	0.4472	4.5 × 10^5^	0.2038	9.8 × 10^5^	0.0001	2.2 × 10^9^
	Dietary	UB	13.1955	1.5 × 10^4^	0.0039	5.1 × 10^7^	0.2353	8.5 × 10^5^	0.4475	4.5 × 10^5^	0.2095	9.5 × 10^5^	0.0008	2.6 × 10^8^
3 to 6 years	Mean	LB	3.9888	5.0 × 10^4^	-	-	0.1713	1.2 × 10^6^	0.1525	1.3 × 10^6^	0.0483	4.1 × 10^6^	0.0006	3.4 × 10^8^
	Dietary	UB	4.0097	5.0 × 10^4^	0.0008	2.5 × 10^8^	0.1716	1.2 × 10^6^	0.1529	1.3 × 10^6^	0.0496	4.0 × 10^6^	0.0012	1.7 × 10^8^
	Extreme	LB	9.8413	2.0 × 10^4^	-	-	0.3899	5.1 × 10^5^	0.6568	3.0 × 10^5^	0.1399	1.4 × 10^6^	0.0058	3.5 × 10^7^
	Dietary	UB	9.8859	2.0 × 10^4^	0.0030	6.6 × 10^7^	0.3908	5.1 × 10^5^	0.6570	3.0 × 10^5^	0.1439	1.4 × 10^6^	0.0106	1.9 × 10^7^
7 to 12 years	Mean	LB	1.7607	1.1 × 10^5^	-	-	0.0347	5.8 × 10^6^	0.0945	2.1 × 10^6^	0.0477	4.2 × 10^6^	0.0004	5.4 × 10^8^
	Dietary	UB	1.7738	1.1 × 10^5^	0.0006	3.5 × 10^8^	0.0349	5.7 × 10^6^	0.0948	2.1 × 10^6^	0.0491	4.1 × 10^6^	0.0009	2.1 × 10^8^
	Extreme	LB	4.1957	4.8 × 10^4^	-	-	0.1754	1.1 × 10^6^	0.3508	5.7 × 10^5^	0.0812	2.5 × 10^6^	0.0030	6.6 × 10^7^
	Dietary	UB	4.2303	4.7 × 10^4^	0.0022	9.2 × 10^7^	0.1759	1.1 × 10^6^	0.3510	5.7 × 10^5^	0.0834	2.4 × 10^6^	0.0064	3.1 × 10^7^
13 to 19 years	Mean	LB	1.0089	2.0 × 10^5^	-	-	0.0236	8.5 × 10^6^	0.0961	2.1 × 10^6^	0.0336	5.9 × 10^6^	0.0002	1.1 × 10^9^
	Dietary	UB	1.0173	2.0 × 10^5^	0.0004	4.8 × 10^8^	0.0237	8.4 × 10^6^	0.0963	2.1 × 10^6^	0.0346	5.8 × 10^6^	0.0007	2.8 × 10^8^
	Extreme	LB	2.1918	9.1 × 10^4^	-	-	0.0909	2.2 × 10^6^	0.3108	6.4 × 10^5^	0.0697	2.9 × 10^6^	NA	NA
	Dietary	UB	2.2144	9.0 × 10^4^	0.0016	1.3 × 10^8^	0.0913	2.2 × 10^6^	0.3108	6.4 × 10^5^	0.0717	2.8 × 10^6^	0.0016	1.3 × 10^8^
20 to 64 years	Mean	LB	1.1770	1.7 × 10^5^	-	-	0.0516	3.9 × 10^6^	0.1066	1.9 × 10^6^	0.0578	3.5 × 10^6^	0.0001	1.5 × 10^9^
	Dietary	UB	1.1846	1.7 × 10^5^	0.0004	5.6 × 10^8^	0.0519	3.9 × 10^6^	0.1068	1.9 × 10^6^	0.0595	3.4 × 10^6^	0.0007	2.9 × 10^8^
	Extreme	LB	1.4470	1.4 × 10^5^	-	-	0.1995	1.0 × 10^6^	0.2809	7.1 × 10^5^	0.1222	1.6 × 10^6^	NA	NA
	Dietary	UB	1.4667	1.4 × 10^5^	0.0014	1.4 × 10^8^	0.2003	1.0 × 10^6^	0.2809	7.1 × 10^5^	0.1255	1.6 × 10^6^	0.0017	1.2 × 10^8^
Over 65 years	Mean	LB	1.5509	1.3 × 10^5^	-	-	0.1158	1.7 × 10^6^	0.0730	2.7 × 10^6^	0.1343	1.5 × 10^6^	0.0001	3.7 × 10^9^
	Dietary	UB	1.5612	1.3 × 10^5^	0.0003	7.4 × 10^8^	0.1161	1.7 × 10^6^	0.0731	2.7 × 10^6^	0.1381	1.4 × 10^6^	0.0004	4.8 × 10^8^
	Extreme	LB	2.1444	9.3 × 10^4^	-	-	0.5716	3.5 × 10^5^	0.1473	1.4 × 10^6^	0.4206	4.8 × 10^5^	NA	NA
	Dietary	UB	2.1700	9.2 × 10^4^	0.0012	1.6 × 10^8^	0.5733	3.5 × 10^5^	0.1473	1.4 × 10^6^	0.4320	4.6 × 10^5^	0.0013	1.5 × 10^8^

^(1)^ Estimated daily intake (ng/kg b.w./day), ^(2)^ NA: food consumption data were not available.

## Data Availability

The original contributions presented in this study are included in the article. Further inquiries can be directed to the corresponding authors.

## Supplementary Materials

The following supporting information can be downloaded at https://www.mdpi.com/article/10.3390/toxins17020050/s1, Figure S1. Optimization of analytical method for MON quantification. Table S1. Food consumption for mean dietary and 95th percentile extreme intake scenarios by age group.
