# Supplementary material for: Assessment of Natural Occurrence and Risk of the Emerging Mycotoxin Moniliformin in South Korea
Source: Toxins (Basel). 2025 Jan 23;17(2):50. doi: 10.3390/toxins17020050 (PMC11860269; doi:10.3390/toxins17020050)
Supplement: Supplementary file 1 [file toxins-17-00050-s001.zip › toxins-3403388-supplementary.pdf]

Supplementary Material

Assessment of Natural Occurrence and Risk of the Emerging Mycotoxin Moniliformin in South Korea

So Young Woo [1], Sang Yoo Lee [1], Su Been Park [1], Si Eun Kim [2], Young Woon Kang [3] and Hyang Sook Chun [1,*]

[1] GreenTech-Based Food Safety Research Group, BK21 Four, School of Food Science and Technology, Chung-Ang University, Anseong-si 17546, Republic of Korea; mochalatte9@naver.com (S.Y.W.); dm3822@naver.com (S.Y.L.); sirius6100@naver.com (S.B.P.)

[2] Department of Food Safety and Regulatory Science, Chung-Ang University, Anseong-si 17546, Republic of Korea

[3] Food Contaminants Division, National Institute of Food and Drug Safety Evaluation, Ministry of Food and Drug Safety, Cheongju-si 28159, Republic of Korea; youngcloud@korea.kr

* Correspondence: hschun@cau.ac.kr; Tel.: +82-31-6703290; Fax: +82-31-6753108

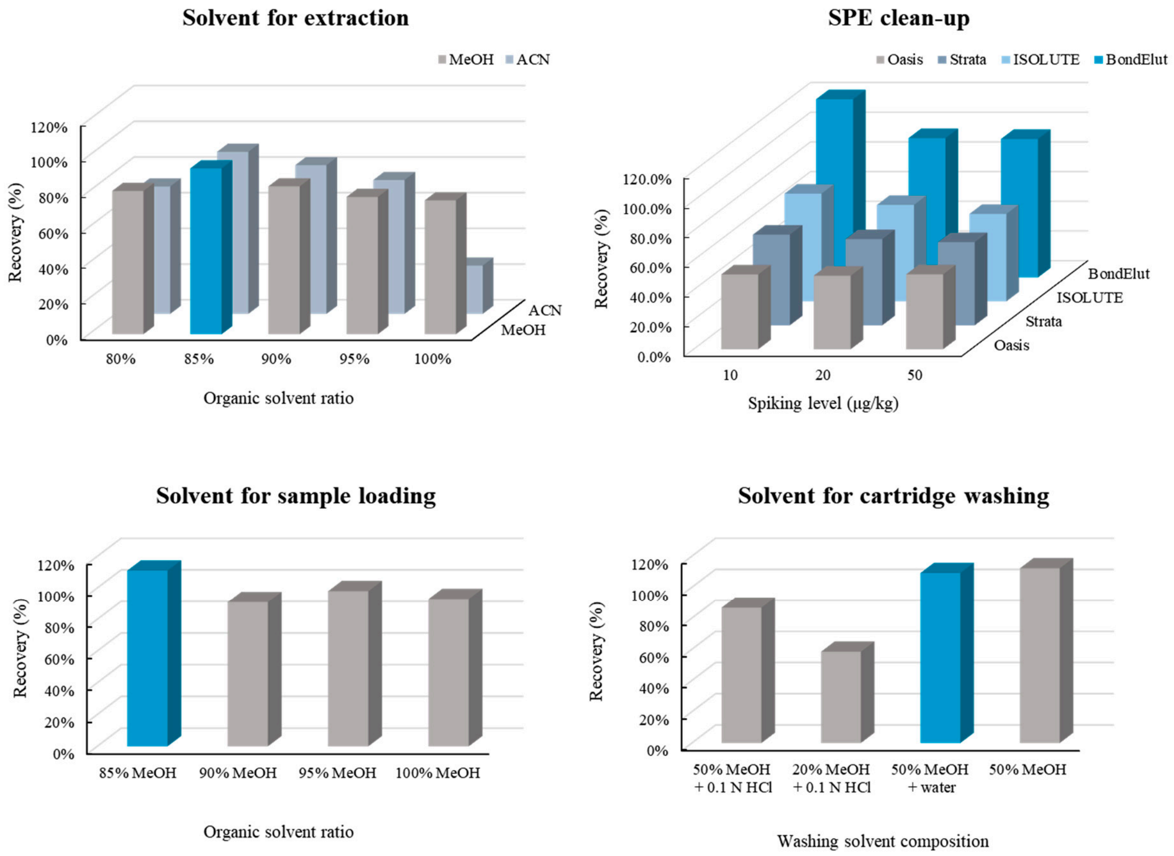

Figure S1. Optimization of analytical method for MON quantification.

Table S1. Food consumption for mean dietary and 95th percentile extreme intake scenarios by age group.

Food commodity	Mean dietary intake (g/day) by age group							Extreme dietary intake (g/day) by age group						
	All ages	1 to 2	3 to 6	7 to 12	13 to 19	20 to 64	Over 65	All ages	1 to 2	3 to 6	7 to 12	13 to 19	20 to 64	Over 65
White rice	127.44	93.31	105.45	127.42	128.51	125.40	142.99	280.40	196.61	187.10	255.27	294.90	284.48	291.28
Brown rice	5.53	1.98	2.77	5.26	6.14	5.19	7.65	28.87	9.93	15.12	27.95	33.71	27.22	38.79
Black rice	6.38	3.28	5.61	10.69	9.20	6.18	4.95	15.52	5.51	32.63	101.11	38.66	13.95	14.18
Barley	5.54	1.56	2.46	4.49	4.44	4.85	10.30	31.02	11.28	15.35	26.24	26.45	27.73	46.52
Oats	0.95	0.42	0.31	0.76	0.86	0.96	1.17	4.17	0.00	0.00	5.62	4.29	3.64	6.55
Job's tears	0.05	0.00	0.02	0.03	0.01	0.05	0.06	0.00	0.00	0.00	0.00	0.00	0.00	0.00
Sorghum	0.31	0.19	0.38	0.36	0.31	0.28	0.44	1.09	1.45	2.10	2.00	1.96	0.32	2.22
Maize	4.87	4.43	5.14	3.61	3.06	4.97	5.62	2.25	9.65	10.00	4.75	1.50	2.15	0.00
Foxtail millet	0.49	0.19	0.38	0.51	0.47	0.44	0.74	2.56	0.73	3.31	3.31	2.78	2.22	3.58
Wheat flour	5.51	2.04	4.15	4.66	7.22	6.16	2.91	34.16	10.59	24.36	26.05	36.64	35.95	17.00
Canned maize	0.56	0.51	0.84	1.05	1.11	0.58	0.04	0.00	0.39	0.54	0.61	0.00	0.00	0.00
Popcorn maize	0.13	0.05	0.07	0.26	0.29	0.13	0.01	0.00	0.00	0.00	0.00	0.00	0.00	0.00
Sunsik	0.75	0.09	0.16	0.17	0.38	0.88	0.69	0.00	0.00	0.00	0.00	0.00	0.00	0.00
Red bean	0.41	0.08	0.09	0.17	0.39	0.38	0.73	0.00	0.17	0.23	0.23	0.52	0.00	0.00
Mung bean	0.13	0.07	0.06	0.11	0.07	0.12	0.21	0.00	0.00	0.00	0.00	0.00	0.00	0.00
Black bean	6.00	1.97	1.34	2.65	2.97	5.55	11.63	14.63	3.64	3.91	4.54	6.19	11.78	36.55
Red pepper powder	2.06	0.14	0.35	1.02	2.03	2.39	1.55	8.00	0.61	1.89	4.45	7.65	8.76	6.09
Curry powder	0.51	0.32	0.69	0.87	0.66	0.53	0.20	0.00	0.07	6.81	7.13	0.00	0.00	0.00
Turmeric	0.01	0.00	0.00	0.00	0.00	0.01	0.00	0.00	0.00	0.00	0.00	0.00	0.00	0.00
Nutmeg	0.00	0.00	0.00	0.00	0.00	0.00	0.00	0.00	0.00	0.00	0.00	0.00	0.00	0.00
Peanut	0.64	0.29	0.12	0.20	0.20	0.68	0.90	1.03	0.28	0.21	0.14	0.00	1.05	2.88
Walnut	0.31	0.03	0.08	0.08	0.06	0.34	0.45	1.02	0.00	0.20	0.05	0.00	1.23	2.91
Almond	0.62	0.14	0.19	0.23	0.15	0.71	0.65	2.70	0.01	0.49	0.16	0.00	3.00	4.07
Sesame seed	0.69	0.25	0.31	0.56	0.72	0.72	0.70	2.58	0.86	1.25	2.61	3.33	2.64	2.48
Perilla seed	0.29	0.11	0.27	0.11	0.11	0.27	0.55	1.18	0.23	0.62	0.56	0.46	1.07	2.77
Corn oil	0.10	0.01	0.10	0.11	0.15	0.10	0.06	0.15	0.05	0.12	0.17	0.17	0.17	0.08
Sesame oil	1.39	0.76	0.85	1.20	1.15	1.52	1.17	5.64	3.11	3.29	4.78	4.60	6.01	5.13
Perilla oil	0.34	0.13	0.17	0.23	0.17	0.33	0.57	1.47	0.60	0.60	0.90	0.68	1.38	3.07
Red pepper seed oil	0.02	0.00	0.00	0.02	0.04	0.02	0.01	0.00	0.00	0.00	0.00	0.00	0.00	0.00
Grapeseed oil	0.13	0.12	0.11	0.18	0.28	0.12	0.06	0.00	0.12	0.12	0.03	0.00	0.00	0.00
Rapeseed oil	0.43	0.35	0.52	0.49	0.57	0.44	0.27	2.14	1.56	2.93	2.30	2.52	2.30	1.51
Olive oil	0.38	0.13	0.34	0.58	0.55	0.39	0.22	1.25	0.53	1.33	2.56	2.71	1.33	0.41
Soybean oil	3.31	1.66	2.43	3.49	4.94	3.66	1.36	15.02	7.93	9.39	14.84	20.96	15.80	7.42
